# Widowhood and grandchild care: a longitudinal study of European grandmothers and grandfathers

**DOI:** 10.1007/s10433-026-00908-x

**Published:** 2026-02-20

**Authors:** Elisa Tambellini, Mirkka Danielsbacka, Antti Olavi Tanskanen, Hans Hämäläinen, Anna Rotkirch

**Affiliations:** 1 Population Research Institute of Väestöliitto, Helsinki, Finland; 2 INVEST Research Flagship Centre, University of Turku, Turku, Finland

**Keywords:** Grandparental care, Widowhood, Sex differences, SHARE data

## Abstract

The death of a spouse is a major life event, commonly experienced in later adulthood. While existing research suggests that widowhood may reduce the provision of grandchild care, most studies have relied on cross-sectional comparisons between widowed and non-widowed individuals. In this study, we use longitudinal data from the Survey of Health, Aging, and Retirement in Europe (SHARE; *n* = 27,467) to examine how changes in partnership status are associated with grandparental caregiving over time. Using panel fixed effects regression models, we find that widowhood significantly decreases grandchild care provision among grandfathers but not among grandmothers. For grandfathers, grandchild care declines by approximately 13 days per year shortly after widowhood, 19 days 2 years after, and 16 days 4 years after, relative to pre-widowhood levels. In contrast, the effects for grandmothers are small and statistically non-significant. These findings suggest that in contemporary Europe, grandfathers substantially reduce their caregiving involvement following the loss of a spouse, whereas grandmothers’ provision of grandchild care remains largely resilient to widowhood.

## Introduction

In contemporary families, grandparents are increasingly active in their grandchildren’s lives throughout childhood. Increased longevity and declining fertility have enhanced the duration grandparental caregiving, particularly among women (Pasqualini et al. 2021; Zamberletti et al. 2018; Chapman et al. 2017). Compared to earlier generations, present-day grandparents are generally healthier, wealthier and have fewer grandchildren, so they can spend more time with each grandchild (Arber et al. 2012; Pasqualini et al. 2021). Grandparental childcare has many beneficial effects on the younger family generations. It can enhance adult children’s fertility (e.g., Rutigliano 2024; Tanskanen et al. 2014a, b), boost grandchild wellbeing (e.g., Helle et al. 2024), and increase mothers’ labor force participation (Aassve et al. 2012; Yu et al. 2023). Moreover, research has shown that active grandparenting—when not too intense—can substantially improve the health and wellbeing of grandparents themselves (see Danielsbacka et al. 2022, for a review). Notably, the grandparental ‘happiness bonus’, or increase in life satisfaction associated with caring for grandchildren, is especially large among widows compared to those in stable marriages (Rotkirch et al. 2024). Thus, close relations with grandchildren can be a factor of individual resilience that helps to adjust to the loss of a spouse. However, at the same time, not all grandparents may have the same capacities or possibilities to continue grandparenting after losing a spouse.

Widowhood is a major life course transition typically occurring in older age that alters the dynamics of extended family relations. While several studies have examined how parental union dissolution affects grandparent-grandchild ties (e.g., Attar-Schwartz et al. 2009; Lussier et al. 2002; Tanskanen et al. 2014a, b), less attention has been given to the impact of grandparental union dissolution.

Cross-sectional research examining childcare frequency among grandparents across different marital statuses (married, remarried, divorced, and widowed) has revealed consistent patterns (e.g., Danielsbacka and Tanskanen 2016; King 2003; Knudsen 2012). Divorced and remarried grandfathers typically demonstrate reduced caregiving engagement. In contrast, grandmothers maintain higher involvement regardless of marital status when compared to grandfathers (Danielsbacka and Tanskanen 2016). Some research even indicates that divorced grandmothers provide more childcare than their married, widowed, or repartnered counterparts (Perry and Daly 2021).

We wish to contribute to this body of research by examining the change in partnership status and its effect on grandchild care from a longitudinal perspective following the same individuals over time. *Previous longitudinal work has examined grandparental childcare and its health and wellbeing correlates, and several panel studies have included partnership status as a predictor of caregiving behavior (for example, *Di Gessa et al. 2016; Hong and Xu 2023). *These studies use longitudinal designs to study changes in caregiving intensity or wellbeing over time, and some analyze differences by marital/partnership status. However, most existing analyses either treat widowhood as one of many covariates, focus on wellbeing outcomes rather than the caregiving transition itself, or are not based within-person analyses explicitly comparing caregiving levels before and after spousal loss. To our knowledge, this is the first study to explicitly compare grandchild caregiving behavior within the same individuals’ lives before and after the transition to widowhood.*

Specifically, we examine how the transition to widowhood affects grandparental caregiving, and whether widowed grandmothers continue caregiving at higher rates than widowed grandfathers, as suggested by previous cross-sectional studies. To answer these questions, we employ longitudinal data from the Survey of Health, Aging, and Retirement in Europe (SHARE), using data from 27 European country and Israel.

## Theoretical background

Both kin lineage and a multitude of other socioeconomic, demographic, and geographic factors are known to shape grandchild care provision (for an overview see: Coall et Hertwig 2010). Here, we focus on two basic and consistently important factors: gender and marital status.

### Gender differences in grandparental investment

One of the most common findings in grandparent studies is that grandmothers are consistently more involved in their grandchildren’s lives than grandfathers (Daly and Perry 2017; Coall and Hertwig 2010; Danielsbacka et al. 2011), especially when we compare them within the same kin lineage (e.g., maternal grandmothers to maternal grandfathers) (Tanskanen et al. 2011). Evolutionary scholars have explained this gendered caregiving pattern through the reproductive strategies that shape caregiving behaviors. Women’s higher reproductive costs—such as pregnancy, childbirth, and direct child-rearing—extend into their roles as grandmothers, reinforcing their continued investment in later generations. In contrast, male reproductive strategies have historically focused more on mating compared to parenting, as well as been more facultative and flexible, allowing for less obligatory involvement in caregiving (Euler 2011; Sear and Mace 2008; Trivers 1972). This biological asymmetry, combined with social norms and expectations, helps to explain the gendered patterns of caregiving seen in both parenthood and grandparenthood.

In family sociology, the tendency for women to take on greater caregiving responsibilities compared to men has been explained through kin-keeper theory, which suggests that women are more often than men socialized to maintaining family ties and engaging in caregiving activities (Bracke et al. 2008; Dubas 2001). The rationale behind the kin-keeper theory is often expressed through socialization, so that girls are raised to adopt nurturing roles, while boys are socialized to expect less involvement in family caregiving (Hoffman 1977). These deeply ingrained expectations persist across generations, making women more likely to take on caregiving responsibilities in later life as well (Gerstel and Gallagher 2001; Lawrence et al. 2002). In today’s Europe, grandmothers’ greater involvement is further reinforced by structural factors, such as their longer life expectancy and earlier retirement (Silver 2019), which afford them more time for childcare. Male grandchild care can, in turn, be more affected by breadwinner expectations, higher retirement age, and masculine social styles. Over the last generations, European men have become much more involved in care provision of their children and their grandchildren, as well (Danielsbacka and Tanskanen 2016).

### Marital status and grandparental caregiving

Marital status plays a crucial role in shaping grandparental involvement in caregiving, often intersecting with gender to influence the extent and nature of engagement with grandchildren. Research consistently finds that married grandparents maintain the highest levels of contact with their grandchildren, followed by widowed, remarried, and divorced grandparents (Albertini and Garriga 2011; Perry and Daly 2021 for a different order). The loss of a spouse—whether through widowhood or divorce—typically leads to a decline in caregiving involvement, though previous research suggests this effect is more pronounced for grandfathers than for grandmothers (Knudsen 2012; Danielsbacka and Tanskanen 2016).

One key explanation for this gender difference is the incidental exposure hypothesis (Euler and Michalski 2008), which suggests that grandfathers often engage with grandchildren more passively, with their interactions largely facilitated by their wives, the grandmothers of their shared grandchildren. As a result, the absence of a spouse can significantly disrupt these connections, leading to a marked decline in caregiving involvement for widowed or divorced grandfathers (Knudsen 2012). In contrast, grandmothers are more likely to maintain direct caregiving roles even if they do not have a spouse. Some studies found that grandmothers who do not have a spouse provide more childcare help than their married counterparts (Perry and Daly 2021).

Given that older men are more likely to live with a spouse than older women, they benefit from the stability of marriage for a longer period (Knudsen 2012). However, once widowed, men may experience a steeper decline in caregiving involvement, as they may have relied on their spouse to maintain connections with their extended family. This divergence highlights the importance of considering how marital transitions interact with gender to shape grandparents. While marriage provides structural support for continued caregiving, its loss may affect grandfathers and grandmothers differently. The traditional division of care work within families, where women take on primary caregiving responsibilities while men’s involvement is often secondary, means that grandmothers are better positioned to maintain caregiving roles even in widowhood.

## Aim of the study

While extensive research has examined grandparental caregiving, the specific effects of widowhood on this role have not previously been explored from a longitudinal perspective. Effects that are visible from cross-sectional studies may be modified once we follow changes over time among the same individuals. This study seeks to fill this gap by investigating how the transition to widowhood influences the frequency of grandparental childcare among contemporary European grandparents. Drawing on sociological and evolutionary perspectives, which are complementary rather than mutually exclusive, we will here explore whether grandmothers, as primary kin-keepers, are more likely to continue caregiving than grandfathers, whose involvement may be more dependent on the presence of a spouse. Prior research shows that caregiving responsibilities are unevenly distributed between men and women—grandmothers typically engaging in more hands-on care—and we investigate how this pattern alters with widowhood.

The two research questions are:

**Q1:** Does becoming widowed influence the frequency of grandparental childcare?

**Q2:** Does the effect of widowhood on grandparental childcare frequency differ between grandmothers and grandfathers?

## Data and methods

### Sample

This study uses longitudinal data drawn from the Survey of Health, Aging and Retirement in Europe (SHARE), a large-scale, cross-national panel survey that collects data on individuals aged 50 and older across multiple European countries. SHARE provides detailed information on various aspects of aging, including health, socioeconomic status, and family dynamics, making it a rich source for examining changes in grandchild caregiving over time. The data used in this study span from the first to the nine waves of SHARE, covering the period from 2004 to 2022.

The analytic sample was derived from an initial pool of 146,781 respondents, following a series of inclusion criteria. Participants were included if they (1) had at least one grandchild aged 14 years or younger,^1^(2) were either continuously married (or cohabiting) or transitioned from marriage (or cohabitation) to widowhood during the observation period, (3) had multiple survey observations, (4) had complete data on the grandchild care variable, and (5) had complete information on covariates.

**Fig. 1:**
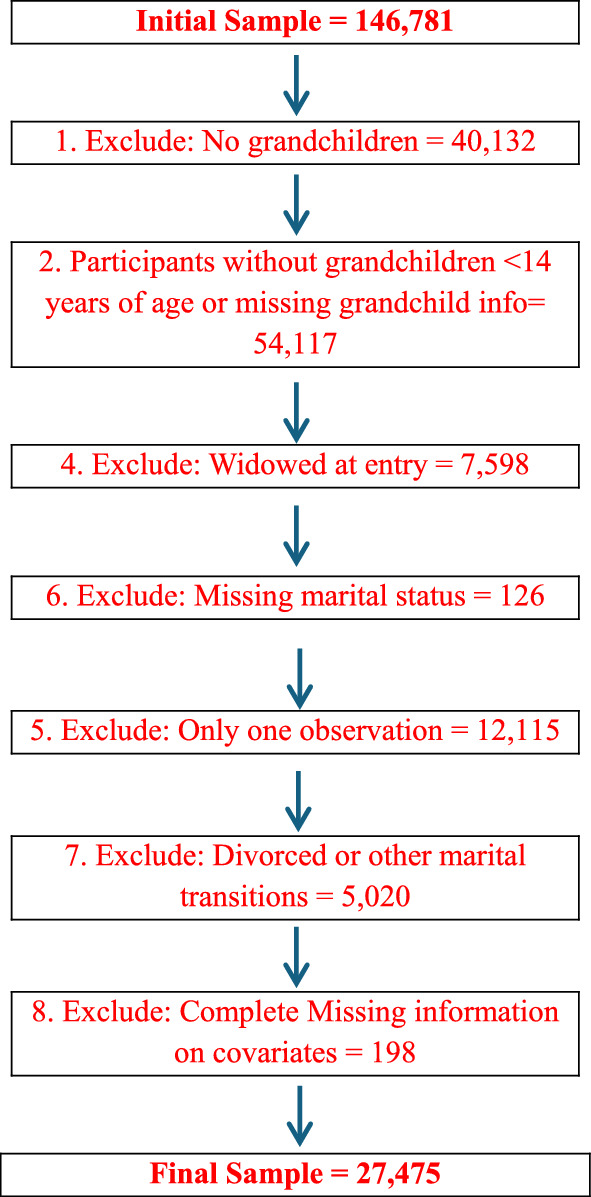
Flowchart of sample selection process

This selection process ensures that the analysis captures the transition from being partnered (either married or cohabiting) to widowhood. For comparison, continuously married or cohabiting individuals of similar ages were also included. A detailed flowchart outlining the sample selection process has been added (see Fig. 1). After applying these criteria, the final analytic sample consisted of 27,475 respondents (of which 53% are female), and we observed 2490 widowhood transitions (see Table 1). The fixed effects models were estimated using the 27,467 individuals who contributed a total of 68,693 person-wave observations.

**Table 1:** Descriptive statistics at the baseline (first observation) and at the last follow-up wave of observation (only for time-variant variables)

	Men		Women	
	First wave	Last wave	First wave	Last wave
% Education				
Isced 0&1	21.3	–	22.3	–
Isced 2	15.2	–	19.3	–
Isced 3&4	40.5	–	39.6	–
Isced 5&6	23.0	–	18.7	–
% Country area of residence				
Northern Europe	13.3	–	11.8	–
Southern Europe	18.9	–	19.1	–
Central Europe	34.7	–	30.4	–
Eastern Europe	28.4	–	33.9	–
Israel	4.8	–	4.8	–
% Retired	61.8	82.0	41.4	63.0
% Self-perceived health				
Less than very good	70.8	79.2	72.7	79.9
% Days of care for grandchildren				
None	38.5	53.0	29.7	46.2
Less than monthly	11.7	9.2	11.7	9.4
About monthly	16.6	13.8	17.0	14.1
Weekly	18.2	13.6	21.7	16.2
Several times per week	15.0	10.4	19.9	14.1
% Widowhood transitions	0	5.0	0	12.6
Mean, (std):				
Age	64.6 (0.7)	71.2 (0.7)	61.5 (0.7)	68.3 (0.6)
Average days of care for grandchildren	58.7 (1.1)	39.7 (1.0)	77.3 (1.2)	52.7 (1.1)
Number of grandchildren	3.6 (0.3)	4.3 (0.3)	3.7 (0.3)	4.4 (0.3)
Number of grandchildren < 15 years of age	1.6 (0.0)	1.4 (0.0)	1.6 (0.0)	1.4(0.0)
Age of youngest grandchild	3.8 (0.3)	9.3 (0.3)	3.8 (0.3)	9.4 (0.3)
N	12,891		14,584	

### Dependent variable

The dependent variable in this study is grandchild care, measured by respondents’ self-reported involvement in caring for their grandchildren. SHARE participants who had at least one grandchild were asked whether they had provided care for their grandchild(ren) without the parents being present, either during the time interval since the last interview (for follow-up waves) or during the preceding 12 months (for new entrants to the survey). If respondents indicated that they had provided care, they were asked to report how often they had done so. The frequency of care was measured on a four-point scale: 1 = Almost every day, 2 = Almost every week, 3 = Almost every month, and 4 = Less often. We converted the scale to represent the number of days spent on grandchild care annually (0, 6, 12, 52, and 365 days). When respondents reported care for multiple grandchildren, caregiving frequency was first recorded separately for each grandchild and then summed across grandchildren to obtain the total number of caregiving days per respondent per wave. After converting the scale, researchers summed up the days of care provided to all children of their offspring and then capped the value at a maximum of 365 days per year (Hämäläinen et al. 2025).^2^

### Independent variable

The main independent variables examined in this study is time to widowhood. Respondents are asked to describe their current relationship status from the options: “married,” “registered partnership,” “never married,” “divorced or separated,” and “widowed.” Respondents are classified as widowed if they select this option. With this information, we constructed a variable measuring the time to widowhood, i.e., considering the time before and after the year of partner’s death. The literature investigating major life events emphasizes the importance of examining anticipation and adaptation to them (Frijters et al. 2011; Kung 2020). It is also likely that spousal death was preceded by at least 1 year of worse health, and that the year after spousal death was especially heavy for the wellbeing of the widow. Therefore, time to widowhood was measured through five dummy variables: two or more waves before widowhood t >  = − 2; one wave before widowhood *t* = − 1; shortly after widowhood *t* = 0; the wave after widowhood *t* = 1; and two or more waves after widowhood t >  = 2.^3^

### Covariates

Several factors known to affect grandchild care provision were controlled for in the data analysis. Sociodemographic variables that could change across waves were treated as time-varying, whereas all time-invariant factors were controlled by the design in the individual fixed effects models (e.g., education for people over 50; country of residence; personality traits). The time-varying covariates included age (in years), employment status (retired vs. not retired), number of grandchildren under 15 years of age, age of the youngest grandchild, and overall self-rated health status. Health status was assessed using a four-point scale (1 = very good, 2 = good, 3 = fair, 4 = poor) and dichotomized into "very good" versus "less than very good”. The distributions of these variables at the sample baseline (first observation) and at the last observation in the study are presented in Table 1.

Some other factors also influencing grandchild care, notably geographical distance, kin lineage, and sex of grandchild, were not included here since they are characteristics of the grandchild, and our sample size in the widowed category did not allow for fine-grained analyses between different types of grandchild clutches.

### Analytic strategy

We first employed nonparametric kernel-weighted local polynomial plots to illustrate the continuous trajectories of grandchild care provision from 3 years before to 3 years after spousal death. These plots allow us to visualize changes in grandchild care around widowhood, comparing both widowed individuals and their non-widowed counterparts. For widowed individuals, the plots are centered around the wave corresponding to spousal death, while for non-widowed individuals, the plots are aligned with the average age at which spousal death occurred for individuals which experienced widowhood (see Kung 2020).

Next, we used individual fixed effects regression models to estimate how grandchild care changes with widowhood status and duration. Fixed effects models were selected for their suitability with longitudinal data, as they control for time-invariant variables that might confound the relationship between social resources and subjective wellbeing. These models are ideal for examining within-individual changes over time, accounting for characteristics like country of residence, and baseline traits such as personality and levels of wellbeing, which could otherwise distort the results (Allison 2009).

The outcome variable, grandchild care frequency, is a continuous one, reflecting caregiving levels across time. We first estimated a model that included all time-varying covariates listed above, stratified by sex, and then plotted the predicted values to visually highlight gender differences in caregiving roles. The main explanatory variable was the categorical indicator of time relative to widowhood, divided into five categories, as described above. For individuals in intact marriages, this variable remains constant, serving as the control group. Including this category, rather than focusing solely on widowed individuals, offers a key advantage, since they may also experience changes in grandchild care, even without widowhood, due to age or ill health, for instance. Excluding them would risk overestimating the impact of widowhood on caregiving (Bayaz-Ozturk et al. 2018; Streeter 2020).

Although the sample is unbalanced, as participants have varying numbers of repeated measurements, all individuals were observed at least twice—once before and once shortly after widowhood.

## Results

### Descriptive characteristics of the sample

Descriptive statistics for all time-variant variables included in the analyses are shown in Table 1, stratified by gender. Respondents were on average in their early 60 s at the beginning of the study, with men slightly older compared to women. Health perceptions were relatively similar for both sexes, with nearly 30% of participants rating their health as very good or excellent. Education distribution was also relatively balanced between genders. On average, participants had around 3.6 grandchildren, with 1.6 grandchildren under the age of 15 and the youngest grandchild on average 4 years old.

Regarding the distribution of grandchild care frequency at the baseline, a significant proportion of grandparents provided no care at all, with this trend being more pronounced among men than women. Among those who did provide grandchild care, the most common frequency was "weekly" followed by "several times per week". Women were more likely than men to engage in frequent caregiving, highlighting the expected gendered division of grandparental roles. Indeed, grandmothers provide, on average, about 20 more days of care per year than grandfathers at the baseline. Finally, a larger proportion of women experience widowhood during the observational period compared to men.

### Widowhood and grandchild care

**Fig. 2:**
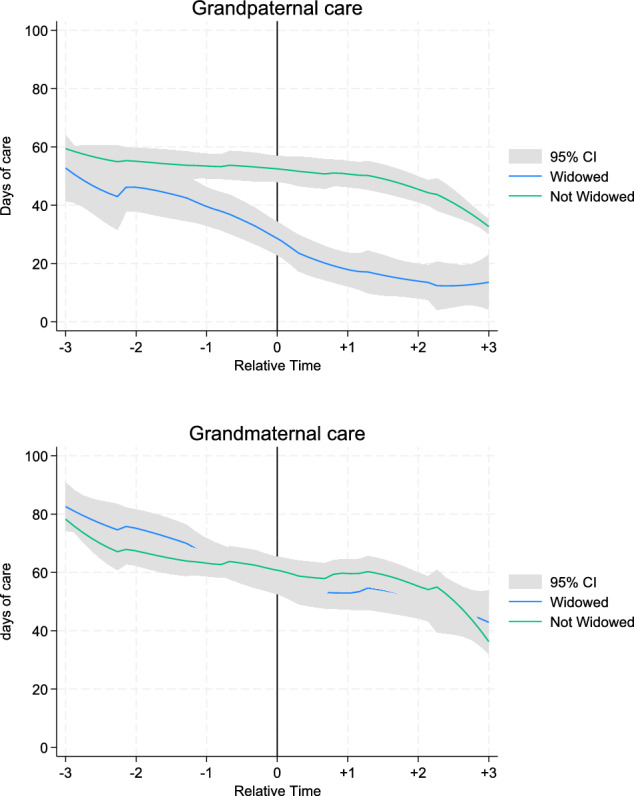
Trajectories of days of grandchild care per year before and after spousal death, comparing widowed and non-widowed grandmothers and grandfathers (95% CI). Note: The blue curve is a local polynomial smoothed plot of days of care per year in the widowed sample by waves since spousal death, with the vertical line representing the wave of spousal death. The green curve plots days of care per year in the non-widowed sample, with the vertical line representing the average age at which spousal death occurred for those who experience widowhood (73 years of age for men and 70 for women)

We first analyze how widowhood is associated with the caregiving frequencies of grandparents over time. Figure 2 illustrates the trajectories of grandchild care provision before and after spousal death for respondent who experience spousal death and for those who did not, using nonparametric polynomial smoothed plots stratified by sex. Both grandfathers and grandmothers spent fewer days caring for grandchildren as time progresses, regardless of their widowhood status. This suggests, as expected, that aging itself plays a role in reducing caregiving responsibilities.

For grandfathers, the data show a pronounced decline in grandchild care following spousal death (time 0). Widowed grandfathers experience a substantial reduction of approximately 30 days of care per year within the first 3 years after spousal loss, decreasing from around 45 days before widowhood to about 15 days afterward. In contrast, non-widowed grandfathers show a much more gradual decline over the same period, maintaining relatively stable levels of around 45–50 days of care per year before slowly tapering off to around 30–35 days.

For grandmothers, the pattern is different. Both widowed and non-widowed grandmothers demonstrate a decline in grandchild care over time, but the difference between the two groups is much smaller. Widowed grandmothers show a decrease of about 20–25 days over the 3-year span, from roughly 80-day pre-widowhood to about 55–60-day post-widowhood. However, non-widowed grandmothers also show a similar decline, suggesting that widowhood has only a modest additional impact on grandmaternal caregiving. The confidence intervals for widowed and non-widowed grandmothers largely overlap throughout the observation period.

Overall, these graphs indicate that spousal death is associated with a stronger reduction in grandchild care for grandfathers than for grandmothers, both in absolute terms and relative to their non-widowed counterparts. This highlights a notable gender difference in the impact of spousal loss on grandparental caregiving involvement.

The fixed effects regression models further explore the association between widowhood and the number of days of grandchild care, stratified by gender. Table 2 quantifies the effect. For males, widowhood is associated with a significant reduction in grandchild care. Shortly after becoming widowed, grandfathers provide approximately 13 fewer days of care per year (*p* = 0.017), and this reduction becomes even more pronounced one wave after widowhood, with a decrease of about 19 days (*p* = 0.009). The negative effect persists two or more waves after widowhood, with a reduction of approximately 16 days (*p* = 0.029). These results suggest that spousal death has a sustained and statistically significant impact on grandfathers’ involvement in caregiving over time.

**Table 2:** Effect of widowhood for grandparents´ care to grandchildren (dependent variable: days of care per year in categories), by sex of grandparents

	Grandfathers	Grandmothers
Time to widowhood (ref. 2+ waves before widowhood)		
1 wave before widowhood	− 4.12	1.00
Shortly after widowhood	− 13.03*	− 0.21
1 wave after widowhood	− 18.89**	3.19
2+ waves after widowhood	− 15.96*	− 0.34
Age	− 1.24***	− 2.59***
Self-rated health < very good	0.40	− 1.85
Retired	20.20***	14.99***
At least one grandchild < 15 years old	11.35***	12.54***
Age of youngest grandchild	− 1.55***	− 1.06**
N	12,891	14,584
Obs	31,493	37,200

t statistics in parentheses: * *p* < 0.05, ** *p* < 0.01, *** *p* < 0.001

For females, widowhood appears to have no significant impact on the number of days of grandchild care. Across all widowhood timing categories (before, shortly after, 1 wave after, and 2+ waves after), the coefficients are small and statistically non-significant.

Together, these results show that widowhood substantially reduces caregiving among grandfathers but not among grandmothers, reinforcing the gendered patterns observed in the descriptive figure.

Beyond widowhood status, other key variables also shaped caregiving patterns. Age negatively influenced caregiving for both men and women, as suggested also by the Fig. 2. Retirement, on the other hand, was associated with increased caregiving for both grandfathers and grandmothers. Having more grandchildren under the age of 15 was positively correlated with increased caregiving responsibilities, while the increase in age of the youngest grandchild was associated with decreased grandchild care provision.

**Fig. 3:**
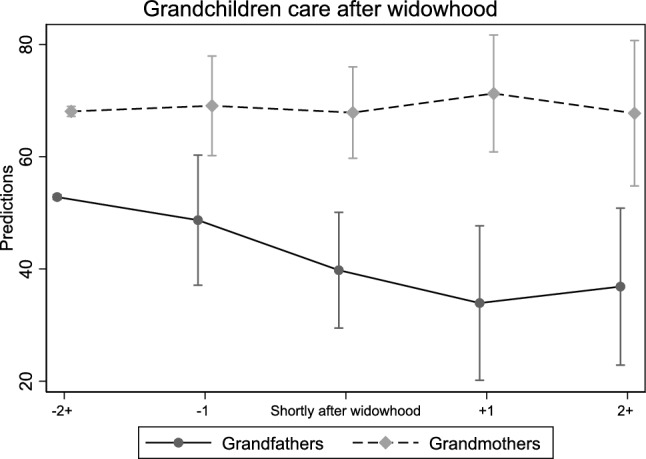
Marginal effects of grandchild care (days per year) during widowhood, by sex of grandparents. 95% C.I. Note: − 2+ : two or more waves before; − 1: one wave before widowhood; + 1: one wave after; > 2+ : two or more waves after

Figure 3 graphically illustrates gendered differences in grandchild caregiving before and after widowhood, plotting the marginal predicted values of days of grandchild care per year. Women’s caregiving patterns appear largely stable across the widowhood transition. Predicted caregiving days remain consistently around 65–70 days per year, with minimal variation between pre- and post-widowhood periods. Among grandfathers, while caregiving levels are already lower than those of women before widowhood, predicted caregiving days decrease steadily from approximately 50 days per year before widowhood to about 30–35 days per year one wave after spousal death. A slight, non-significant rebound is visible two or more waves after widowhood, but caregiving remains well below pre-widowhood levels. The results suggest a decline in the time spent providing grandchild care over the observation period. However, this pattern should be interpreted cautiously, as the confidence intervals for the estimates overlap considerably, particularly around the event period and even at later time points. Moreover, the span between the earliest and latest observations (approximately 8–10 years) represents a substantial aging period for participants entering the study at around age 70, which may partly account for the observed decline.

**Fig. 4:**
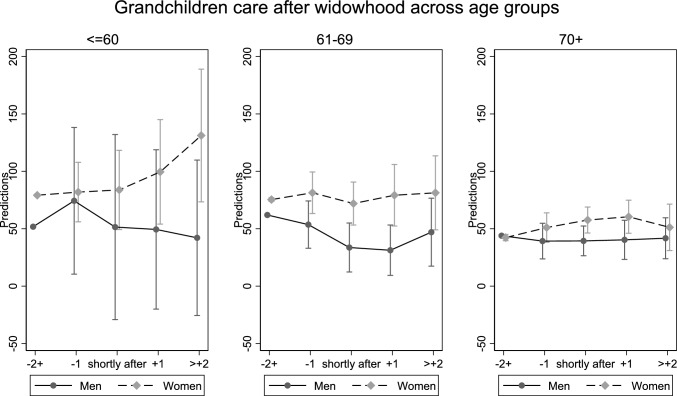
Marginal effects of grandchild care (days per year) during widowhood, by sex and age of grandparents. 95% C.I. Note: − 2+ : two or more waves before; − 1: one wave before widowhood; + 1: one wave after; > 2+ : two or more waves after

**Fig. 5:**
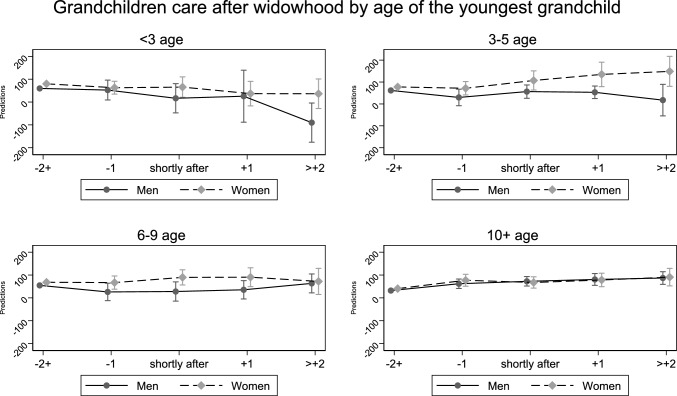
Marginal effects of grandchild care (days per year) during widowhood, by sex of grandparents and age of grandchild(ren). 95% C.I. Note: − 2+: two or more waves before; − 1: one wave before widowhood; + 1: one wave after; > 2+ : two or more waves after

To better examine the role of age in the caregiving process, we conducted a robustness check by stratifying the analysis by the age of both grandparents (≤ 60, 61–69, 70 +) and grandchildren (< 3, 3–5, 6–9, ≥ 10). The results, presented in the Appendix (Figs. 4 and 5), show no substantive differences compared with the main models.

**Fig. 6:**
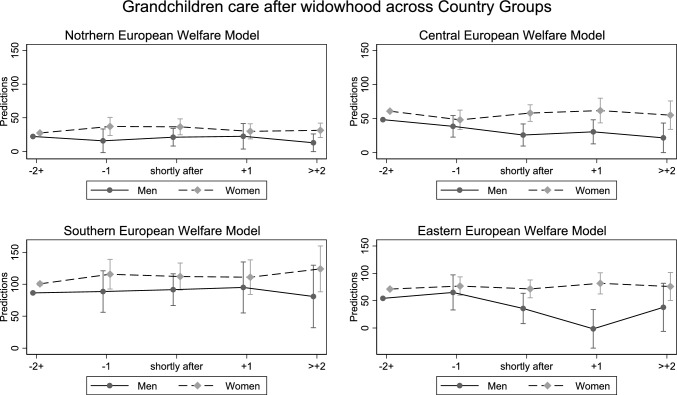
Marginal effects of grandchild care (days per year) during widowhood, by sex of grandparents and Country area. 95% C.I. Note: − 2+ : two or more waves before; − 1: one wave before widowhood; + 1: one wave after; > 2+ : two or more waves after

As an additional robustness check, we also examined caregiving patterns by country groups: Southern countries (Greece; Italy; Spain; Portugal; Cyprus; Malta); Northern countries (Sweden; Denmark; Finland); Eastern countries (Czech Republic; Poland; Hungary; Slovenia; Estonia; Croatia; Lithuania; Latvia; Bulgaria, Romania; Slovakia); and Central European countries (Austria; Germany; Netherlands; France; Switzerland; Belgium; Luxembourg). This stratification was motivated by the expectation of substantial cross-country heterogeneity in spousal mortality and health, age at grandparenthood, and grandchild care provision across Europe. The results (Appendix, Table 3; Fig. 6) indicate that while the gendered response to widowhood appears relatively consistent across contexts, the institutional and cultural frameworks of different country regimes shape both the intensity of grandparental care and the capacity of grandfathers to maintain caregiving after losing a spouse.

## Discussion

We examined the impact of widowhood on grandchild caregiving among European grandparents that were followed over time. Our findings suggest that the transition to widowhood significantly alters caregiving patterns, particularly for grandfathers. Widowed grandfathers exhibit a sharp and sustained decline in grandchild care provision, which was almost halved during the 4 years following the loss of a spouse.

Our analysis reveals clear gender differences in grandparental caregiving at baseline across all respondents. These findings support the incidental exposure hypothesis (Euler and Michalski 2008), which proposes that in contemporary high-income societies, grandfathers’ caregiving is often mediated through their spouses. When widowed, grandfathers experience a significant reduction in caregiving engagement as this marital connection is severed. In contrast, grandmothers maintain more consistent caregiving levels after widowhood, suggesting their caregiving role is more intrinsic and less dependent on marital status.

These results reinforce established gender differences in grandparental investment from complementary theoretical perspectives. Sociological frameworks emphasize how gender socialization and cultural expectations position women as primary caregivers throughout life (Gerstel and Gallagher 2001; Lawrence et al. 2002). Evolutionary perspectives suggest grandmothers’ greater caregiving investment stems from sex-specific reproductive strategies, while men typically prioritize conjugal relationships (Smith 1991; Euler and Weitzel 1996).

While all respondents showed decreased grandchild care over time—likely reflecting the aging of both grandparents and their grandchildren (with respondents having fewer than four grandchildren on average)—widowhood accelerated this decline, particularly among men. These patterns align with previous research showing women more consistently maintain caregiving responsibilities even without a spouse (Bracke et al. 2008; Danielsbacka and Tanskanen 2016; Dubas 2001; Perry and Daly 2021). The emotional and social disruption following spousal loss appears to contribute to grandfathers’ withdrawal from family roles, while grandmothers more often sustain engagement with both child and grandchild generations. Additionally, adult children may be less inclined to request childcare assistance from widowed grandfathers, even when they are willing and capable caregivers.

This study has some limitations. First, more refined cross-cultural comparisons may reveal whether these patterns hold in non-European contexts, where family structures and cultural norms differ, and where grandparents more often co-reside with grandchildren. Unfortunately, due to the limited number of widowed individuals in our sample, we were unable to conduct multi-level analyses incorporating macrolevel indicators of regional variations in the effects of widowhood on grandparental investment. Future studies should address this limitation by using larger samples and multi-level analyses to better understand country-specific and regional dynamics.

Another key factor to consider is that women’s investment in their grandchildren during widowhood may be influenced by the reduction of caregiving responsibilities they previously had for their spouse. Again, our dataset contained too few cases of caregiving responsibilities before the spouse’s death, limiting our ability to account for this factor. Future research should examine this relationship further to validate our findings.

Methodologically, this study also has limitations related to the two-way fixed effects approach. While fixed effects models help control for unobserved heterogeneity, they may introduce bias when treatment effects are heterogeneous or occur at different times (de Chaisemartin and D’Haultfœuille 2020; Goodman-Bacon 2021; Sun and Abraham 2021). Future research would benefit from employing alternative methods, such as difference-in-differences estimators that account for treatment effect heterogeneity (Roth 2024).

Finally, future studies should examine how changes in grandchild caregiving due to widowhood affect wellbeing. Grandparental care in moderate amounts is associated with benefits in both physical and mental health. This effect is especially clear for widows, who tend to have lower wellbeing and life satisfaction than their married counterparts, but who can compensate for this with other close social relations (Tambellini et al. 2025).

To conclude, our findings also contribute to understanding the gendered consequences of widowhood more broadly, particularly regarding adaptation and mental health. The literature shows mixed results on gender differences in widowhood adjustment: some studies suggest men adapt more quickly to spousal loss (e.g., Siflinger 2017), while others find women demonstrate greater resilience (e.g., Streeter 2020). Our results on caregiving patterns may help contextualize these seemingly contradictory findings. The stability of grandmothers’ care provision suggests continuity in a meaningful social role that may buffer against some negative consequences of widowhood by maintaining social integration and purpose. Conversely, grandfathers’ sharp withdrawal from caregiving may compound other challenges of spousal loss, potentially contributing to social isolation and depression. This loss of a significant family role, combined with the disruption of the spousal relationship that may have facilitated their caregiving engagement, could help explain why some studies find poorer adaptation outcomes among widowed men. The relationship is likely bidirectional: while role loss may contribute to depression, declining mental health following widowhood may also reduce capacity and motivation for caregiving. The resilience of grandmothers in maintaining caregiving roles even after spousal loss is likely to help them adjust to their new life circumstances. However, there is also a need for systems that recognize and, when necessary, alleviate the caregiving burden on older women. Since widowhood affects grandfathers’ caregiving roles as well, more efforts are needed to understand how society can encourage continued grand paternal involvement. Programs that foster social connections and facilitate engagement with grandchildren could help mitigate the decline in caregiving among widowed men.

## Conclusion

This study underscores the significant impact of widowhood on grandchild caregiving, revealing stark gender differences in caregiving trajectories also when studying the same grandparents over time. While grandfathers experience a pronounced and lasting decline in grandchild care after widowhood, grandmothers continue to provide care at relatively stable levels. Understanding the interplay between widowhood, gender, and intergenerational caregiving is essential for addressing the evolving dynamics of family support in aging societies.

## Data Availability

The data underlying this article are available at https://shareeric.eu/data/data-access.

## Appendix

See Tables 3, 4 and Figs. 4, 5, 6.

**Table 3:** Effect of widowhood for grandparents´ care to grandchildren (dependent variable: days of care per year in categories), by sex of grandparents

	Grandfathers	Grandmothers
Time to widowhood (ref. 2+ waves before)		
1 wave before widowhood	− 0.06	− 0.08
Wave of widowhood	− 0.28***	− 0.09
1 wave after widowhood	− 0.27**	− 0.10
2+ waves after widowhood	− 0.47***	− 0.15
Age	− 0.03***	− 0.03***
Health (ref. Very good)		
Less than very good	− 0.03	− 0.03
Retired (ref.no)	0.39***	0.20***
Number of grandchildren younger than 15	0.29***	0.28***
Age of youngest grandchild	− 0.02***	− 0.02***

Note:* t* statistics in parentheses: * *p* < 0.05, ** *p* < 0.01, *** *p* < 0.001

**Table 4:** Descriptive statistics by Country area

	Northern	Central	Southern	Eastern
% Male	49.9	50.2	46.6	42.6
% Widowhood event	9.1	7.7	9.9	10.0
Mean:				
Age of respondent	70.7	69.7	71.5	67.7
Number of grandchildren < 15 years old	1.5	1.4	1.4	1.4
Age youngest grandchild	9.4	9.61	9.7	9.1
Days of grandparental care per year	20.7	42.1	72.6	48.1
N	3,430	8,927	5,215	8,596

## Footnotes

1 If all grandchildren aged into the 15 + category between waves, subsequent observations for that respondent were coded as missing for the grandchild care variable, as no eligible grandchildren remained. This ensures that the analysis reflects only periods in which respondents had at least one potentially care-eligible grandchild.

2 As a robustness check, we also developed a new ordinal variable to measure the total number of days of grandchild care provided: 0 = 0 days (None); 1 = fewer than 12 days (Less than monthly); 2 = 12–51 days (Approximately monthly); 3 = 52–103 days (Approximately weekly); and 4 = 104–365 days (Several times a week). The results using this specification are substantially similar to those obtained with the continuous variable (see Table 3 in the Appendix).

3 SHARE data are collected biennially, meaning each wave represents a 2-year period (e.g., *t* >  = − 2 corresponds to 4 or more years before widowhood; *t* = − 1 corresponds to 2 years before, and so on).
